# Freshmen Program Withdrawal: Types and Recommendations

**DOI:** 10.3389/fpsyg.2017.01544

**Published:** 2017-09-21

**Authors:** Ana Bernardo, Antonio Cervero, María Esteban, Ellian Tuero, Joana R. Casanova, Leandro S. Almeida

**Affiliations:** ^1^Facultad de Psicología, Universidad de Oviedo Oviedo, Spain; ^2^Centro de Investigação em Educação (CIEd), Universidade do Minho Braga, Portugal

**Keywords:** university, undergraduate student, performance, dropout, persistence

## Abstract

University program dropout is a problem that has important consequences not only for the student that leaves but also for the institution in which the withdrawal occurs. Therefore, higher education institutions must study the problem in greater depth to establish appropriate prevention measures in the future. However, most research papers currently focus primarily on the characteristics of students who leave university, rather than on those who choose to pursue alternative courses of study and therefore fail to take into account the different kinds of abandonment. The aim of this paper is to identify the different types of dropout to define their characteristics and propose some recommendations. Thus, an *ex post facto* study was carried out on a sample of 1,311 freshmen from a university in the north of Spain using data gathered using an *ad-hoc* designed questionnaire, applied by telephone or an online survey, and completed with data available in the university data warehouse. A descriptive analysis was performed to characterize the sample and identify five different groups, including 1. Students persisting in their initiated degree 2. Students who change of program (within the same university) 3. Students transferring to a different university 4. Students enrolling in non-higher-education studies 5. Students that quit studying. Also, data mining techniques (decision trees) were applied to classify the cases and generate predictive models to aid in the design of differentiated intervention strategies for each of the corresponding groups.

## Introduction

Higher education withdrawal—including college and university because of their common environmental characteristics—is a largely studied phenomenon in consequence of its implication for the individual, the educational institution and the society (Cope and Hannah, 1975; Pascarella et al., 1986; Duque, 2014). Therefore, professors, stakeholders, and politicians from many disciplines have attempted to study this problem, usually from one of the four most extended paradigms; economic (Jensen, 1981; Di Pietro, 2006; Belloc et al., 2011), psychological (Marín et al., 2000; Peralta et al., 2006; Naranjo, 2009), sociological (Pincus, 1980; Braxton et al., 2000), organizational (Kamers, 1971; Bean, 1983), or educational (Cabrera et al., 2006). In addition, in 1975 Vincent Tinto published his explanatory model of university attrition, being considered as a markland because of its inclusive approach and stated as an example in this research field. Tinto's perspective entails not only the need of assuming a holistic approach to study dropout (taking in regards different kind of factors, ex. economical, sociological, educational, institutional, etc.) but also the need to understand withdrawal as a process in which one is possible to act (Tinto, 1998). Since then, there has been a gradual increase in the institutional attention paid to this phenomenon. In Spain, our research base, this attention has proliferated to a greater extent since the publication of the Royal Decree 1947/1995, which established the National Plan for University Quality, urging universities to evaluate both the processes and results of teaching.

It is necessary to understand than when a student enters a degree, usually have the intention to complete it, but sometimes—for different reasons—can change his or her opinion and take a distinct paths, corresponding to different withdrawal profiles (Andrews et al., 2014): the student can transfer to another program remaining in the same institution (transfer to another degree); he or she could also choose to change of institution (transfer to another institution); there are also some students that opt for lower educational levels (ex. vocational training, on accredited courses); and last, some students resolve quit studying.

Therefore, governments and institutions have the responsibility to look into the dropout problem deeply, taking in consideration its different types while analyzing the roots and particularities of this phenomenon; this knowledge is a value a base for the design of intervention measures able to decrease dropout rates, in spite of their handicaps of budget and personnel. This, in turn, leads to important savings for both the university and the students alike, as in Spain the annual cost of academic abandonment surpass 1,500 million Euros (Colás, 2015). Apart from economic cost, Higher Education institutions are affected by their dropout rates at deeper levels as well; their efficacy is called into question, and this can have negative effects on faculty motivation as well as on its enrollment rates (Angulo-Ruiz and Pergelova, 2013; Hossler and Kalsbeek, 2013). Furthermore, a country's national development is often impacted by Higher Education Professional graduation and employability rates; therefore, increases in dropout levels could slow the national development pace. Although these consequences exemplify the high cost of withdrawal, the students, and their families undoubtedly face the worse part as they have to deal not only with the financial loss but also with a new extremely challenging decision process about their future (González et al., 2007; Arriaga et al., 2011).

Hence, the diagnosis of the problem turns into an investment that, if accompanied by the application of preventive and corrective actions, can generate great benefits for every part involved (Colás, 2015). Nevertheless, current research that is developed using exclusively secondary data (available on the university information systems) might be insufficient to establish effective preventative measures, since it ignores important dimensions of the problem. Thus, studies using primary data instead would be much more advisable. Among this type of methodology, there are two major tendencies regarding the kind of information examined:

On the one hand, there are studies aiming to distinguish the characteristics of students who quit, compared with those who remain in the institution (these studies are the most common, as a smaller sample is needed to provide generalizable results). Identifying the differential characteristics of the students who withdraw, allows establishing prevention measures. Nevertheless, unless the diverse types of abandonment are differentiated, preventive strategies will obtain uneven results (Bernardo et al., 2015).

On the other hand, some researchers intend to distinguish between the diverse types of abandonment. The close examination of these profiles of dropout can serve as a base of an early alert system by means of the identification of student risk factors, and ultimately lead to the application of specific intervention strategies in the future (La Red-Martínez et al., 2015; Bernardo et al., 2017b). The project implemented by Arriaga et al. (2011) is the most outstanding current example of this type of research in Spain. The authors interviewed (approximately) one thousand students at the Polytechnical University of Madrid and categorized them into seven separate student profiles, and subsequently proposed specific intervention strategies for each kind of dropout.

Clearly, studying Higher Education dropout is methodologically challenging, as often brings along limitations, being the most common either to be based on basic data recorded by the university system—considering only a limited number of variables but obtaining significant results (Bernardo et al., 2017a)—, or to require a large investment of money on surveys to obtain detailed results—acquiring clusters of students and particular conditions but with a lower statistical significance—. Unfortunately, this decision is most often beyond the control of the researchers themselves (Bernardo et al., 2015).

As several authors have already defined the longitudinal (Willcoxson, 2010) and temporal (Tinto, 1988) dimensions of Higher Education withdrawal, we can conclude that this process can start as early as the time that schooling begins (Bernardo et al., 2017b). Therefore, students often enter university with very different backgrounds (Rumberger, 1983; Bedard, 2001; Crawford, 2014) and personalities (Heilbrun, 1965; Pandey, 1973; Alkan, 2016), which produces a broad casuistry that must be explored in depth by taking into account each student's point of view (Tinto, 2015).

In this sense, the emerging technology of Educational Data Mining creates new opportunities, as it is able to make sense of a large amount of data and find patterns that are difficult to identify with inferential statistics alone (Romero and Ventura, 2013). Data mining compiles the knowledge produced by both Statistics and Artificial Intelligence, while at the same time remaining accessible to educators (Hand, 1998; Baker and Yacef, 2009). In regard to Higher Education dropout, three of the possible techniques have proven helpful to understanding the problem, since they are used to raise and solve classification problems in which a certain number of variables are used as predictors (acting as a criterion variable); these techniques are association rules (López et al., 2015; Badr et al., 2016), Naive Bayes (Moseley and Mead, 2008; Moreno-Salinas and Stephens, 2015; Shaleena and Paul, 2015) and Decision Trees (Escobar et al., 2016; Hasbun et al., 2016; Liang et al., 2016).

Of these techniques, decision trees were considered the most appropriate for the present study, as they fit our phenomenon character by being able to explain a subject's behavior when confronting a decision, and (data permitting) reflect the longitudinal process associated with the decision (Yasmin, 2013; Nagrecha et al., 2017). The analysis output provides a network of nodes, which show how the dependent variable behaves regarding the rest studied variables. However, since data mining techniques are optimal mainly for a large amount of data and their application to analyzing dropout patterns is not widespread, there are few examples of their use in current literature. Therefore, the present study intends to contribute in this sense, considering that Yasmin (2013) has already demonstrated that decision trees are optimal to identify patterns of learner attrition.

The present paper attempts to apply these techniques to analyze a university student sample collected within the framework of *The Alfa-GUIA Project* for a Comprehensive Management of University Dropout (funded by the European Commission, DCI-ALA/2010/94). The Alfa-GUIA project aims to address the Higher Education dropout phenomenon from a holistic approach. To do so, its aims and actions are based on four strategies: First, to understand the problem by means of an extensive review of both literature and international research; Second, to assess and spread good prevention practices; Third, to promote greater integration in educational policies and, Fourth to engage the different agents involved (Proyecto Alfa-GUIA, 2014a).

Twenty-one higher education institutions took part in the project and collaboratively developed a questionnaire, which was subsequently completed in sixteen of them. Thus, nearly ten thousand students from all over the world participated in the international study. The global results can be consulted on the Alfa-GUIA web page as well as in the official reports (http://www.alfaguia.org; Proyecto Alfa-GUIA, 2014b). The analysis of this paper focuses on a medium size Spanish university that participated in Alfa-GUIA study. The Alfa-GUIA questionnaire included two common blocks to be answered by every subject and other specific blocks for each possible alternative academic pathway. These two blocks aim to examine the different dimensions of student experiences and backgrounds which the literature has found to be closely related to increased Higher Education withdrawal levels: sociodemographic variables (Di Pietro, 2006), cultural background (Ghignoni, 2017), economic status (Belloc et al., 2011), institutional related variables (Tinto, 2012), academic behavior (Hasbun et al., 2016), and academic experience (Tinto, 1998).

Our research team, stated the following research question: Is it possible to find a model able to predict dropout regarding a given set of variables? Taking into account previous findings, we hypothesize that (1) Student academic situation cannot be predicted only using secondary data (from the University warehouse) and (2) Academic progress is a variable present in the model. Next section explains the applied research method in detail, to provide a framework for the results and conclusions.

## Method

### Research design

This paper applies the most extended dropout definition in Spain, identifying “dropout students” as those having started a particular university program and decided to do not re-enroll during two subsequent academic years (Cabrera et al., 2006). We assume this definition also following the criteria of most governmental bodies across the world (Arriaga et al., 2011), recognizing its potential to disguise between dropouts and stop-outs (students that take a gap year, once they have initiated their university studies).

An *ex post facto* research design was deemed to be the most suitable, given the characteristics of the phenomenon and the specified dropout definition. As for the variables included in the analysis, we set as the criterio variable the students' academic situation. This variable included five possible values: (1) Students persisting in the initiated university program, (2) Students transferring to another program within the same university, (3) Students transferring to a different university (same or different program), (4) Students transferring to lower educational levels, and (5) Students quitting studies altogether. Therefore, we intend to analyze the main characteristics associated to four of the different kinds of withdrawal (groups 2–5), as well the one corresponding to those that persist (group 1); confirming statistical significant difference between groups of students could contribute to understand the profile associated with each group and increase the efficiency of student affair policies.

As for the variables included in the study, it is necessary to highlight that it focused mainly on those corresponding to block 0 and 1 of the Alfa-GUIA questionnaire regarding dropout and persistence decisions (Proyecto Alfa-GUIA and Grupo de Análisis, 2014).

### Sample

The original sample was comprised of 1,311 subjects, including 700 students that persisted in their initial university program and 611 students that quit their program (95% confidence level and a 3.3% of sample error for both groups). The participants entered our institution in the academic year 2008/9 (40.3%), 2009/10 (43.4%), 2010/11 (13.9%), or 2011/12 (2.4%), as the research team had the intention of using only the first two cohorts but needed to include the last two in order to complete the programmed interviews. The survey process was developed between April and July 2013. We applied a stratified random sampling procedure, regarding the knowledge areas defined by UNESCO (2004), see Table 1.

**Table 1:** Sample distribution regarding UNESCO (2004) knowledge areas.

	**Frequency**	**Percentage**
Education	298	23.0
Arts and Humanities	76	5.9
Social Science, Trade, & Law	308	23.7
Science	124	9.6
Building & Engeniering	296	22.8
Agriculture	20	1.5
Salud y Servicios Sociales	139	10.7
Servicios	37	2.9
Total	1,298	100.0

In regard to the main characteristics of our sample, it is necessary inform that 61.2% of them were 17 or 18 years old, entering university straight away from High School without any delay (no course repetition or gap years, etc.), and reflect a quite balanced participation between men and women (43.1 and 56.9%, respectively). Their socioeconomic characteristics, 93.9% of them were single, 5.2% were married or live with a partner, and the 0.9% were divorced or widow/widower. Only the 41% of their fathers and 36.8% of their mothers hold a Higher Education Diploma.

Nonetheless, subjects who had not answered all the questions we excluded from the analysis, reducing the sample to 697 students who had persisted, and 601 students who had not persisted in their initial program (*N* = 1,298). At this point, it is necessary to clarify that Alfa-GUIA global analysis used a randomized selection of our cases, as it only requires a dropout sample of 541 students and a control group of 174 (Proyecto Alfa-GUIA, 2014b).

### Instrument and procedure

Two procedures were used to gather the data; First, university enrollment services provide us with personal, sociodemographic, and academic information about the students, and second, through the Alfa-GUIA questionnaire completed via email or telephone interview.

As mentioned, the research instrument used was the Alfa-GUIA questionnaire regarding dropout decisions and causes (Proyecto Alfa-GUIA and Grupo de Análisis, 2014). This questionnaire was collaboratively created by all participating institutions and was composed of five blocks: Blocks 2–4, are blocks aiming to a particular student profile that found being useful only for qualitative analysis, far from our current purposes; consequently item from this blocks were excluded from our analysis (Proyecto Alfa-GUIA, 2014b).

Block 0, which included 14 items answered by the institution about student academic profiles and their sociodemographic background, three kinds of variables were measured, including; student sociodemographic background (Cabrera et al., 2006; Trevizán et al., 2009; Belloc et al., 2011), institutional and program characteristics (Tinto, 1975; Braxton et al., 1997; Vries et al., 2011), and student progress in the initiated program (Montmarquette et al., 2001; Willcoxson, 2010; Goldenhersh et al., 2011; La Red-Martínez et al., 2015).

In addition, a large set of variables were provided directly by the students (Block 1) by completing a survey aiming to facilitate a comprehensive analysis of the phenomenon, including six different categories of data: 32 questions about their personal life, culture, economy, university experience, and opinions of several institutional features included as result of an extensive literature review and discussion among the partners of Alfa-GUIA project:

- First, student family background and personal context: Several studies have demonstrated that factors in a student's immediate environment marital status (Di Pietro, 2006), immigration status (Smith and Naylor, 2001), or parental educational level (Rodrigo et al., 2012) might affect their progress in university studies. Thus, this category of variables was included in the present study.
- Second, despite the recent emergence of the “Global Village” phenomenon which tends to decrease discrepancies between cultures (Suárez-Orozco and Qin-Hilliard, 2004; Meneses, 2011; Ghignoni, 2017), cultural characteristics continue to play a role in the dropout phenomenon and were therefore considered in the study.
- The Alfa-GUIA study also considered economic variables to be a key research factor, not only regarding family outcome but also in terms of governmental and institutional support: Belloc et al. (2011), among other authors, has demonstrated that this dimension still plays a remarkable role in Higher Education dropout levels.
- Institutional variables (such as size, infrastructure, and human resources) as well as those specifically related to the program of study and its faculty (including program length and the amount of effort required, quality of teachers or services provided) have also been closely linked to the phenomenon and defined as key factors in prevention measures (Cuseo and Farnum, 2011; Tinto, 2012) Therefore, the present work has deeply examined the student perception of both the institution and the program in great depth.
- Student academic behavior, regarding class attendance, participation in extracurricular activities, time devoted to study, etc. is often associated with academic results and decisions regarding persistence (Trevizán et al., 2009; Santos and Vallerado, 2013; Hasbun et al., 2016).
- The last category included in the study was related to the student academic experience, seeing as how this variable could potentially influence both dropout decisions as well as compound other dimensions of the problem (Pascarella and Terenzini, 1983; Tinto, 1998; Wilcox et al., 2005; Gilardi and Guglielmetti, 2011; Pluut et al., 2015).

### Data analysis

Descriptive and decision tree (exhaustive CHAID) analysis were performed with IBM Statistical Package for the Social Sciences, version 22. A decision tree is a predictive data mining technique that creates a classification model based on flow charts, which then allows for the classification of cases and the prediction of criterio variable values regarding the predictive variables included in the analysis (Berlanga et al., 2013), see Table 2.

**Table 2:** Decision tree specifications and results synthesis.

**Specifications**							**Results**			
**Method**	**Dependent variable**	**Independent variable**	**Validation**	**Tree deep**	**Min cases entry nod**	**Min cases exit node**	**Included independent variables:**	**Number of nodes**	**Terminals**	**Depth**
Exhaustive chaid	Academic status	Every variable included in Block 0 and 1 were included as independent variables:	None	3	100	50	Academic progress (%of passed credits regarding the program overall credits), Entry age, Time devoted to studying and kind of housing during the academic year	15	9	3

*Method: Exhaustive Chaid. Dependent variable: academic situation*.

## Results

**Figure 1:**
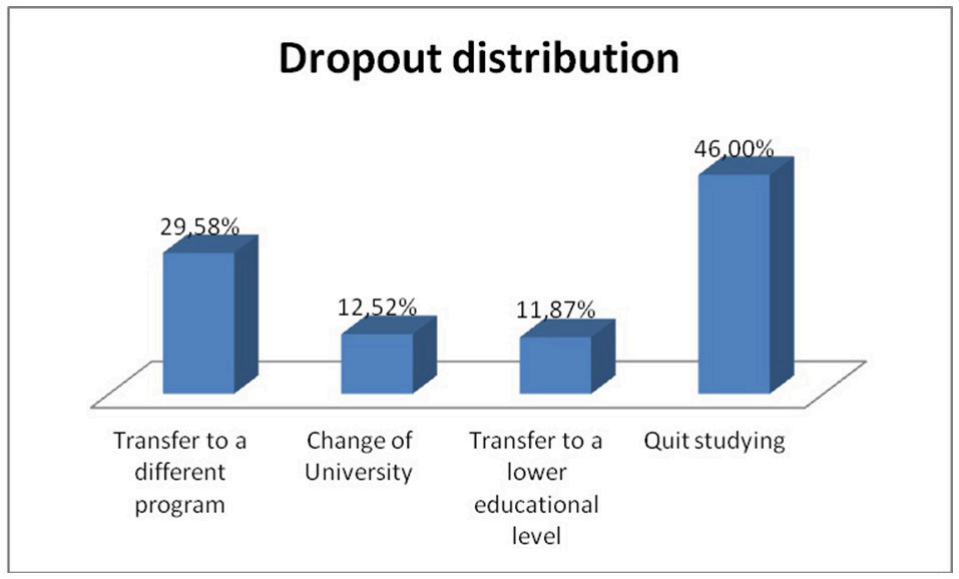
Dropout subsample composition.

The sample was composed of students both students who had persisted in their initial university program (53.7%) and students that had quit (46.3%). This second group includes several kinds of dropout, as shown in Figure 1.

However, the options “transfer to another university” and “transfer to a lower educational level” represent a low contribution to this subsample, which is why they have been excluded from the tree analysis. Therefore, after having omitted these two values, a decision tree representing the academic situation (including persistence in the initiated degree, transfer to a different program and quit studying altogether) of the students was built which proved to obtain acceptable classification values.

**Figure 2:**
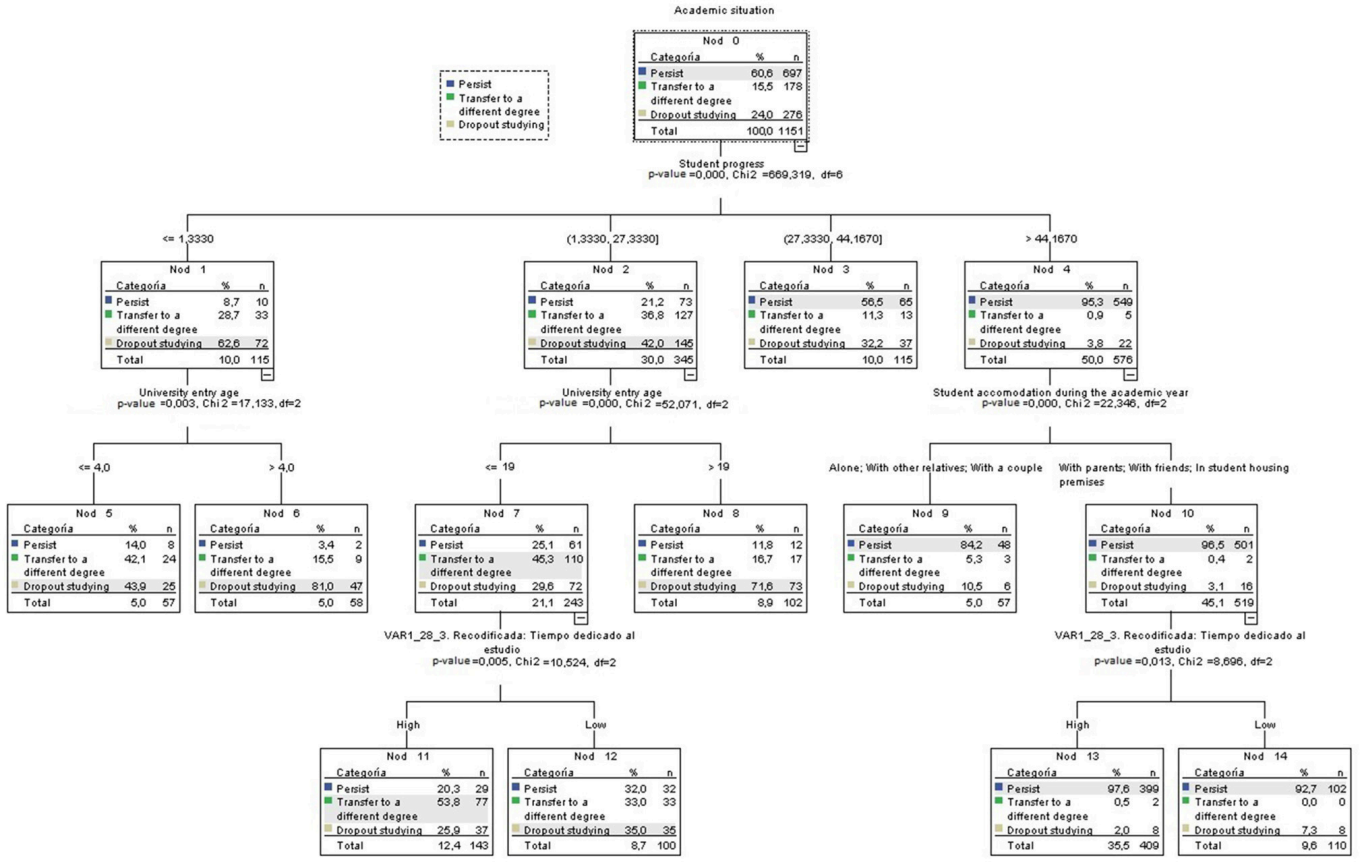
Decision tree.

As observed in Table 3, el 75.7% of the subjects were classified, being the model particularly accurate for groups 1—persistence in the initial university program—and 3—quit studying—, with an accuracy of 88.1 and 65.2%, respectively. However, this value is notably lower for group 2—transfer to a different program within the same university (43.3%). However, the latter remains relevant, as the model shows a similitude between the features of students in group 1 and 2 (see Figure 2), which will be discussed further in the next section.

**Table 3:** Confusion matrix.

**Observed**	**Prognosticated**			
	**Persist on the initial degree**	**Transfer to a different program**	**Quit studying**	**Correct percentage**
Persist on initial degree	614	29	54	88.10
Transfer to a different program	18	77	83	43.30
Quit studying	59	37	180	65.20
Overall percentage	60.00	12.40	27.50	75.70

Figure 2 shows how student progress is the variable that most predicts the academic situation of the students for each of the three groups, being the percentage of passed credits overall the programs' (χ^2^ = 669.319; *p* = 0.000). In this sense, had passed more than the 44.16% of program's credit classify 95.3% of the students that persist, decreasing its classification potential for lower performance intervals. Thus, as academic progress decreases, the probability of dropout increases.

On a second level, the group containing students with lower student progress (<27.33% of overall program credits) is influenced by the age at the time of university entry. The tree in Figure 2 shows two different situations; first, those with a student progress level equal or lower to 1.33% (χ^2^ = 17.133; *p* = 0.003), for students who are 20 years old or younger at the time of enrollment, is associated with lower proportion of dropout and transfer paths (43.9 and 42.1%, respectively). Conversely, enrolling at age 20 or older leads to a higher percentage of dropout cases (81%). Therefore, it can be concluded that a low student progress in addition to entry at an older age can lead to higher dropout, while having a low student progress and a younger age (age 20 or under) tends to result in a higher proportion of students choosing to redirect their educational path by means of degree transfer (42.1%), rather than to quit studying (43.9%). Something similar happens in the group with a student progress level between 1.33 and 27.33% (χ^2^ = 52.071; *p* = 0.000), even though in this case the entry age cutoff would be lower: where students over age 19 at the time of university entry tend to quit 71.6% of the time, and those aged 19 or younger are more likely to transfer to another degree and even more if we take in regard time devoted to study. In this last case, devoting a large amount of time to studying is linked to higher rate of degree transfer, whereas the results are uneven in cases that report having devoted a little time to studying regardless of the group being considered.

Lastly, among the students that have shown the highest level of student progress (having passed at least the 44.16% of their program credits), are classified regarding their type of residence (χ^2^ = 22.346; *p* = 0.000). Specifically, living with parents, friends or in special student accommodation also increases the probability of academic persistence (96.5%), although the amount of time devoted to studying also remains a relevant factor. Students that report having devoted a lot of time to studying tend to persist in their initial program of study (97.6%), as opposed to those that declare to have devoted a little time. In contrast, students who live alone, with other relatives but parents or with their partner, have a higher probability of dropping out, doing so 10.5% of the time and persisting in their studies 84.2% of the time. Therefore, living with parents, friends or in student accommodation facilities can help to prevent student withdrawal.

**Figure 3:**
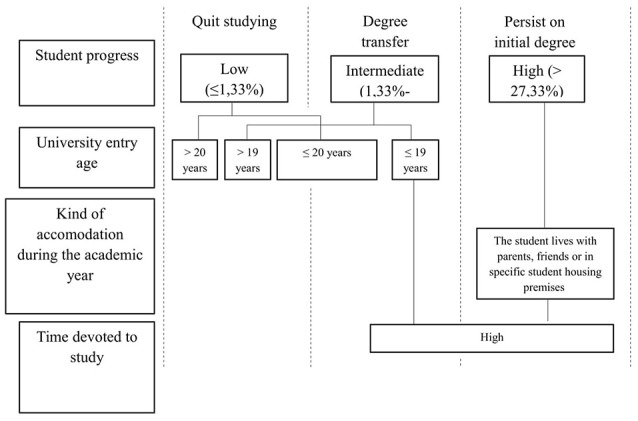
Persistence, Program Transfer, and Dropout Profiles.

In light of these results, the student profile for each of the studied groups can be defined as follows (Figure 3).

As reflected in Figure 3, students that persist are characterized by a good progress in the program (having passed more than the 27.33% of its overall credits), live with their parents, friends or in specific student housing premises and consider that they devote a high amount of time to study. Students that opt to transfer to other degree present a lower academic progress (between 1.33 and 27.33% of the program credits passed) are 19 or fewer years old and also consider that they devote a high quantity of time to study. Last, students that quit studying present a really low progress (having passed 1.33% or less of the program credits) and tend to entry university with older age (20 years or more).

## Discussion

The previous Figure 3 illustrates the relationship of the student group related to the academic status, and the variables influencing decisions regarding academic persistence^1^, providing a student profile characterizing each group. These profiles provide valuable information for the institutional decision-making process:

- First, the group of students deciding to quit studying after having started a university program is mainly composed of older students with lower levels of academic progress. Therefore, institutions should establish specific measures for this mature and low-performance students to increase their persistence rates (Rubin and Wright, 2015; Mountford-Zimdars et al., 2017).
- Second, the group of students transferring to another program is mainly composed of students with low to intermediate level of academic progress but who show a high level of dedication to their studies. As stated by Andrews et al. (2014) this profile is harder to characterize, as there are different reasons to transfer and a wider range student processes underlying them. Consequently, we cannot equalize those freshmen that enter a particular program with the idea of transferring (as they could not match their preferred degree entry requirements) to those that transfer as a result of institutional persistence policies or a mismatch between their expectations and the programmed reality.
- And third, the group of students persisting in their degree is characterized by having higher levels of academic progress and living with their parents, friends or in student housing (with a partner or other relatives). Although this profile of students shows an appropriate adaptation to the program requirements, it is also necessary to establish some intervention measures aiming to secure their engagement and maximize their potential development (Arriaga et al., 2011; Duque, 2014; Quaye and Harper, 2014).

Some similarities were found between the persistence and transfer profiles, which can be understood to be connected to the level of determination of the second group to quit the initiated program, but also to continue their university studies (Arriaga et al., 2011).

Above the 46 studied variables, four of them proved to be the most influential on students' withdrawal decisions: Student progress was clearly the most important variable, reflecting the great impact of academic excellence on both policies and student performance. These results are consistent with those which have been obtained by other researchers (Cabrera et al., 2006; Willcoxson, 2010; Belloc et al., 2011; Goldenhersh et al., 2011; Casaravilla et al., 2012; Crawford, 2014; Moreno-Salinas and Stephens, 2015) and suggest to develop educational measures in order to avoid knowledge gaps and promote a better performance (King et al., 2015).

The second most influential variable was the age of the student. The results highlight that younger students entering university straight after High School are more likely to persist in their studies than those who have taken a break (regardless the length) or repeated a year before their entry into university. These findings agree with results obtained by several authors using different methods (Montmarquette et al., 2001; Smith and Naylor, 2001; Di Pietro, 2006; Yasmin, 2013; Soria-Barreto and Zuñiga-Jara, 2014). This results highlight the need that our institution (as many others) have to acquire: older students (often referred as mature or non-traditional students) are increasing their presence in our institutions and require special adaptations (timing, teaching methods, etc.) to match their educational needs (Shepherd and Sheu, 2014). No other variable seems to play a substantial role in student dropout pathways, and moreover these two (academic progress and age) have been linked to the phenomenon not only at university stage, but also in prior academic levels (Brunello and Checci, 2007; Lassibille and Navarro, 2009; Clotfelter et al., 2012; Diaz-Strong and Ybarra, 2016). Perhaps the feeling of failure linked to slow academic progress impacts students' self-esteem (Carabante et al., 2013; Fang and Galambos, 2015) such an extent that it discourages them from studying for a period (Tinto, 2015; Sauvé et al., 2016). This conclusion highlights the importance of promoting student engagement and self-regulation, as it is a variable in which one faculty can act over (Trevors et al., 2016).

In the case of the groups of medium and high academic progress (closely linked to persistence and transfer groups), some additional variables proved to influence their persistence on the institution; Time devoted to studying is the most relevant one, explaining their engagement to the institution by their better academic progress, in comparison to the dropout group (Trevizán et al., 2009; Alarcon and Edwards, 2013; Moulin et al., 2013; Ruiz-Gallardo et al., 2016).

Lastly, and closely linked to students with good academic progress levels, student residence factors (living with parents, friends, or in student housing facilities) stand out as an intermediate variable and contribute to higher levels of academic persistence (Trevizán et al., 2009; Wise, 2013; Moore, 2015). In this sense, Clerici et al. (2015) explain that live-in students spend their day in an environment that motivate them to complete their degrees, meanwhile living with their parents can constraint the time devoted to study as result of the family dynamics, but can also help, as they often act as an external control for the student. Therefore, some kinds of residence can contribute to a better academic progress and, hence, to persist studying (Larsen et al., 2013), making necessary to promote healthy environments to support educational processes (Langford et al., 2011).

## Conclusion

The longitudinal and contextual character of dropout phenomenon makes its study more complex, as many variables are involved in the process and often interact with one to another. Therefore, in light of the results obtained, which have proven to be consistent with other research findings, Data Mining Techniques have proven to be very useful for the present study, as they allow for a more accurate understanding of the complex relationships between the variables (Abu-Oda and El-Halees, 2015; Meedech et al., 2016; Witten et al., 2016). The decision tree illustrating the predictive model shows that each group has certain characteristics in common, which can also serve to disguise them from other groups, responding partially to our research question. Variables included in our tree proceed only from the university warehouse. Therefore our first hypothesis (student academic situation cannot be predicted only using secondary data) was rejected. Although some limitations are found, to know these features is the key to promoting better persistence policies. Some authors even state that Educational Data Mining could base early warning systems aiming to prevent a wide range of problematic educational situations (Márquez-Vera et al., 2016).

Above all, the present study highlights the importance of academic progress on persistence decisions among students prior to the European Higher Education Area (EHEA) implantation, confirming our second hypothesis (academic progress is a variable present in the model). EHEA changed the educational perspective, transferring the focus from teaching to learning, from professors to students and stating the European Credit Transfer System was one credit suppose 10 h of lectures and 15 h of students autonomous work (De Wit, 2015). Also, virtual campuses were promoted not only as a teaching tool but also as a space to develop the transversal computational competencies (Tjong and Prabowo, 2016). Regarding that the study programs developed on EHEA frame has proven to be highly demanding regarding self-regulation skills (Triventi, 2014; Ruiz-Gallardo et al., 2016), it would be recommendable to integrate a follow-up of Higher Education Modernization Agenda and student affair services (European Commission, 2006).

This perspective acquires our time (twenty-first century) as the era of technology, where the traditional boundaries to knowledge acquisition have broken down thanks in large part to computer-based environments. Space, time, and even money are no longer the formidable handicaps to accessing quality education that they used to be; and, as stated by the European Commission (2014), virtual learning environments have the potential to spread knowledge, culture, and participation throughout the world, with tertiary education playing a remarkable role in the process (Cerezo et al., 2017). In addition, such a environments facilitate educational research, as they can be used as a tool to access student data (García et al., 2011). Thus, it is paramount for Higher Education Institutions to include consideration of the student withdrawal phenomenon on their agendas, taking the advantage that e-administration open to universities through the implantation of their virtual campuses.

In addition, our study underlines a previously pointed out trend; the recalled non-traditional students (54.4% of our sample was over 19 years old, and 8.7% is over 25 when they enter the institution), related to those students that do not enter the university straight forward high school, and that often have additional responsibilities (family, work and others) that can challenge their progress on the program (Gilardi and Guglielmetti, 2011; Vossensteyn et al., 2015). Since Higher Education is a public good, it is the responsibility of governments and institutions to promote equal opportunities to access, progress, and graduate in this educational setting. In this sense, virtual campuses have the potential not only to better monitor student progress—as previously commented—but also to be used as a mechanism to fill in knowledge gaps and promote the engagement of this non-traditional students (Van Doorn and Van Doorn, 2014).

## Limitations and further research

As highlighted by Arriaga et al. (2011), to effectively prevent university dropout, it is not enough to act solely during the context in which the dropout occurs, but rather during the previous educational stages as well. To be able to achieve this goal, the creation of a common Data Warehouse would be a great advantage (Miñaca and Hervás, 2013). Not having unlimited access to student data was a limitation of our study, as we could not examine all the available information, neither obtain detail about the transfer to other university profile (which comprises two situations, study the same degree that in the initial institution and start a new degree).

In this sense, Colombia provides a great example of one such warehouse, as it Spadies System^2^ integrates the information and makes it accessible to anyone interested in studying the problem. While Spain does not have such an advantageous information system as of yet, some researchers are proposing simple and economical methods to promote greater institutional understanding of the process.

## Ethics statement

Ethical approval was not required for this study in accordance with the national and institutional guidelines. The applied procedures in the present study were developed in accordance with the ethical standards of the Research Ethics Committee of the University of Oviedo and the Helsinki Declaration of 1975 and 1983.

## Author contributions

AB contributed to the design of the study and data interpretation. As principal author, she coordinated the writing process of the manuscript. AC and ME are Ph.D. students that study the dropout phenomenon across learning environments, and therefore have participated on each phase of this research. ET, JC, and LA have joint the research team once the survey had been carried out but, since then have largely contribute to the analysis and interpretation of data, and consequently to the understanding of the phenomenon. Every author have play a remarkable role on the writing of this article.

### Conflict of interest statement

The authors declare that the research was conducted in the absence of any commercial or financial relationships that could be construed as a potential conflict of interest.

## Footnotes

^1^As explained before, we studied students that persist on their inicial degree in comparison with those that withdrw it but not necessary exit university studies neither quit studying.

^3^http://www.mineducacion.gov.co/sistemasdeinformacion/1735/w3-article-212299.html
